# Comparison of Temperament and Cognitive Function Between Basketball and Baseball Players

**DOI:** 10.1192/bjo.2024.213

**Published:** 2024-08-01

**Authors:** Kun Jung Kim, Doug Hyun Han, Sun Mi Kim, Kyung Joon Min

**Affiliations:** Department of Psychiatry, School of Medicine, Chung-Ang University, Seoul, Republic of Korea

## Abstract

**Aims:**

The purpose of this study was investigating the differences in temperament, personality, and cognitive function among athletes and non-athletes, as well as differences within athlete groups participating in different-paced sports like baseball and basketball.

**Methods:**

A total of 57 professional basketball players, 51 professional baseball players, and 44 non-athletes subjected to temperament and characteristics inventory assessments and computerized neurocognitive function test. One-way analysis of variance (ANOVA) was employed to analyze the average differences in demographic characteristics, temperament, personality traits, and cognitive functions among the three groups, followed by Bonferroni post hoc tests. Comparisons between starters and non-starters within the athlete groups were conducted using the Mann–Whitney U test.

**Results:**

In the analysis of temperament, the basketball and baseball player groups exhibited higher reward dependence and persistence compared with the control group. Additionally, in the assessment of personality traits, both basketball and baseball player groups scored higher in self-directedness and cooperativeness compared with the control group, whereas self-transcendence scores were lower. In cognitive ability assessments, baseball and basketball players outperformed the control group in emotional perception tests. Both baseball and basketball players showed lower card movement counts compared with the control group.

**Conclusion:**

This study compared the differences in temperament, personality, and cognitive abilities between professional basketball and baseball players and non-athletes. These results provide valuable insights into the temperament, personality, and cognitive abilities of professional athletes, contributing important information for athlete development and coaching goals in the future.

